# RETRACTION: Association Between Malnutrition and Surgical Site and Periprosthetic Joint Infections Following Joint Arthroplasty: A Systematic Review and Meta‐Analysis

**DOI:** 10.1111/iwj.70604

**Published:** 2025-04-21

## Abstract

**RETRACTION:** WangC. and LvS. , “Association Between Malnutrition and Surgical Site and Periprosthetic Joint Infections Following Joint Arthroplasty: A Systematic Review and Meta‐Analysis,” International Wound Journal 21, no. 3 (2024): e14520, 10.1111/iwj.14520.38010066 PMC10898411

The above article, published online on 27 November 2023, in Wiley Online Library (http://onlinelibrary.wiley.com/), has been retracted by agreement between the journal Editor in Chief, Professor Keith Harding; and John Wiley & Sons Ltd. Following an investigation by the publisher, all parties have concluded that this article was accepted solely on the basis of a compromised peer review process. The editors have therefore decided to retract the article. The authors did not respond to our notice regarding the retraction.

**RETRACTION:** C. Wang and S. Lv , “Association Between Malnutrition and Surgical Site and Periprosthetic Joint Infections Following Joint Arthroplasty: A Systematic Review and Meta‐Analysis,” International Wound Journal 21, no. 3 (2024): e14520, 10.1111/iwj.14520.38010066 PMC10898411

The above article, published online on 27 November 2023, in Wiley Online Library (http://onlinelibrary.wiley.com/), has been retracted by agreement between the journal Editor in Chief, Professor Keith Harding; and John Wiley & Sons Ltd. Following an investigation by the publisher, all parties have concluded that this article was accepted solely on the basis of a compromised peer review process. The editors have therefore decided to retract the article. The authors did not respond to our notice regarding the retraction.

